# Filaggrin Gene Defects Are Independent Risk Factors for Atopic Asthma in a Polish Population: A Study in ECAP Cohort

**DOI:** 10.1371/journal.pone.0016933

**Published:** 2011-02-18

**Authors:** Joanna Ponińska, Bolesław Samoliński, Aneta Tomaszewska, Filip Raciborski, Piotr Samel-Kowalik, Artur Walkiewicz, Agnieszka Lipiec, Barbara Piekarska, Jarosław Komorowski, Edyta Krzych-Fałta, Andrzej Namysłowski, Jacek Borowicz, Grażyna Kostrzewa, Sławomir Majewski, Rafał Płoski

**Affiliations:** 1 Department of Medical Genetics, Medical University of Warsaw, Warsaw, Poland; 2 Department of Prevention of Environmental Hazards and Allergology, Medical University of Warsaw, Warsaw, Poland; 3 Department of Dermatology and Venereology, Medical University of Warsaw, Warsaw, Poland; Centre de Recherche Public de la Santé (CRP-Santé), Luxembourg

## Abstract

**Background:**

*FLG* null variants of which 2282del4 and R501X are the most frequent in Caucasians are established risk factors for atopic dermatitis (AD) with an effect probably mediated through impairment of epidermal barrier. Among subjects with AD *FLG* defects are also consistently associated with asthma and allergic rhinitis (AR) but it is less clear to what extent these associations are also present independently from skin disease. The aim of the present study was to evaluate the role of 2282del4 and R501X in predisposing to these allergic phenotypes in a Polish population.

**Methodology:**

2282del4 and R501X were typed among 3,802 participants of the Epidemiology of Allergic Diseases in Poland (ECAP) survey, a cross-sectional population-based study using ECRHS II and ISAAC questionnaires, and ambulatory examination.

**Principal Findings:**

The *FLG* null variants were associated with AD (OR = 2.01, CI: 1.20–3.36, P = 0.007), allergic rhinitis (in particular persistent form, OR = 1.69, CI:1.12–2.54, P = 0.011), and asthma (in particular atopic asthma, OR = 2.22, CI:1.24–3.96, P = 0.006). Association with atopic asthma (but not persistent allergic rhinitis) was also present in the absence of AD, (OR = 2.02, CI: 1.07–3.81, P = 0.027) as well as in the absence of AD and history of broadly defined inflammatory skin disease (OR = 2.30, CI: 1.07–4.93, P = 0.03). Association to atopic asthma would have not been found if diagnosis was made by questionnaire only (OR = 1.15, CI: 0.58–2.32, P = 0.8). We did not observe an association between *FLG* variants and allergic sensitizations (P = 0.8) or total IgE. (P = 0.6).

**Conclusions/Significance:**

In a Polish population *FLG* 2282del4 and R501X carriage increases risk for development of AD and atopic asthma (also in the absence of AD or history thereof). This suggests that interventions aimed at restoring epidermal barrier may have a general role in asthma prophylaxis/treatment.

## Introduction

Filaggrin gene (*FLG*) is strongly expressed in the granular cells of the epidermis leading to production of a large precursor protein profilaggrin. In the process of differentiation profilaggrin is proteolytically cleaved into functional filaggrin peptides which bind and collapse the keratin cytoskeleton and subsequently are degraded into hydrophilic amino acids forming the natural moisturizing factor. The N-terminal domain of profilaggrin is likely to have an additional function as it specifically localizes to the nucleus. All these processes are critical for creation of epidermal barrier with appropriate mechanical and biochemical properties [1].

*FLG* null variants are strong risk factor for AD [2], [3]. In Caucasians two such variants are particularly common: 2282del4 and R501X with originally reported carrier rates in general population of ∼2 and 6%, respectively [2]. Both variants result in a complete loss of processed filaggrin due to premature termination codons within the first *FLG* repeat. Whereas several new *FLG* variants have been reported they are substantially less prevalent and qualitatively different with some residual function [4].

Among subjects with AD *FLG* defects are associated with other allergic disease such as asthma and allergic rhinitis (AR) however, it is less clear to what extent these associations are present independently from skin disease [5]. Two meta-analyses concluded that there was no association between *FLG* null variants and asthma among subjects without AD although the ORs from pooled estimates suggested a trend in the direction of association [5], [6]. Regarding association with AR in the absence of AD two studies reported conflicting results: Weideinger et al. [7] found an effect whereas Marenholz et al. did not [8].

Our purpose was to examine in a Polish population association of the 2282del4 and R501X *FLG* loss-of-function variants with AD, asthma and allergic rhinitis.

## Materials and Methods

### Ethics Statement

The study was approved by Ethical Committee of Medical University of Warsaw. All ECAP subjects gave an informed written consent including specific consent to genetic testing. Written consent for anonymous use of DNA was also obtained from subjects undergoing paternity tests whose samples were used to verify population prevalence of R501X.

### Subjects

The study was based on participants of Epidemiology of Allergic Diseases in Poland (ECAP, www.ECAP.pl) living in major metropolitan areas of Poland (Katowice, Wrocław, Lublin, Gdańsk, Warszawa, Poznań and Białystok). ECAP is a continuation of the European Community Respiratory Health Survey II (ECRHS II) and International Study of Asthma and Allergy in Childhood (ISAAC). ECAP includes randomly selected population aged 20–44 y.o. (ECRHS standard) as well as 6–7 y.o. and 13–14 y.o. (ISAAC standard). The recruitment was done by a randomization procedure based on the personal identity number (PESEL). Out of the 25262 subjects who were approached by pollsters, 9446 refused participation (response rate −62.6%). In the present analysis two of the questionnaire's answers were used (i) “Have you ever had asthma?”, (ii) “Have you ever had eczema, atopic dermatitis or other inflammatory skin condition”.

Those who completed questionnaire were invited for an ambulatory examination. Four thousand thirty eight subjects (25.5%) have taken up the offer. The examination included medical history, physical examination, spirometry, PNIF (Peak Nasal Inspiratory Flow) and skin prick tests with 15 allergens: hazel, alder, birch, grasses/grain, rye, Artemisia, plantain, Alternaria, Cladosporium, molds I (Alternaria tenuis, Botrytis cinerea, Cladosporium herbarum, Culvularia lunata, Fusarium moniliforme, Helminthosporium), molds II (Aspergillus fumigatus, Mucor mucedo, Penicillium notatum, Rhizopus nigricans, Serpula lacrymans, Pullularia pullulans), Dermatophagoides pteronyssinus, Dermatophagoides farinae, dog, cat, negative control, histamine.

Concentration of total IgE in serum was determined with reagents of the Phadia CAP System [9] (N = 3440) or the Allergopharma-ELISA-Test [10] (N = 712). The obtained data were presented in IU/ml (international units per mililitre).

The clinical diagnoses of asthma (atopic or non atopic), intermittent allergic rhinitis (i.e. with symptoms present <4 days a week or for <4 consecutive weeks), persistent allergic rhinitis, (i.e. with symptoms present >4 days a week and for >4 consecutive weeks) and atopic dermatitis were based on the International Global Initiative for Asthma (GINA) guidelines [11], ARIA criteria [12], [13], and criteria of Hanifin and Rajka [14], for asthma, allergic rhinitis and atopic dermatitis, respectively. In addition a history of food allergy, drug allergy, insect bite allergy, urticaria, Quincke's oedema or other chronic diseases was obtained. During the examination blood samples were collected.

While analyzing associations between *FLG* variants and allergic disorders comparisons were performed against a group of healthy controls (N = 1865), defined as individuals without any allergic disorder or other chronic disease (including ichtyosis vulgaris) based on performed clinical workup and history. Family history of atopy or other disease(s) was not an exclusion criterion.

Due to faulty blood sample collection (wrong labeling, degradation) the final genetic analysis was carried in 94% of those who underwent medical exam, i.e. 3802 subjects: 951 children 6–7 y.o. (47.6% females), 1054 children 13–14 y.o. (49.2% females) and 1797 adults (60.7% females).

### Genetic analysis

Genomic DNA was extracted from whole blood. Typing for 2282del4 was performed by sizing of fluorescently labeled PCR product on ABI 3130 sequencer, typing for R501X was done by PCR-RFLP with *NlaIII* restrictase (1639 samples) or TaqMan allelic discrimination assay (2323 samples). One hundred sixty samples were typed for R501X by both methods with complete concordance. All these methods were described previously [2], [8], [15]. During typing every positive sample was repeated by reanalysis of DNA from the original stock. Whole screening was blinded to diagnoses.

### Statistical analysis

In the analysis heterozygous and homozygous genotypes were pooled. Statistical significance of differences in genotype frequency among analyzed groups was assessed with chi square test or Fisher's exact test as appropriate. The strength of association was estimated by calculating Odss Ratio (OR) with 95% confidence interval (CI). Given the reports on the role of the *FLG* null variants in predisposing to AD, asthma and AR no correction for multiple testing was applied. Deviation from Hardy-Weinberg equilibrium was assessed by a Chi-square test with one degree of freedom. Total IgE concentration was log transformed to achieve normal distribution and presented means are geometric means (i.e. back transformed). In the analysis of *FLG* status vs. total IgE the adjustment for test-method, sex and age was performed by univariate ANOVA.

In our study we could detect the following effects with the power of 0.8 at alpha = 0.05: asthma–OR = 2.0, allergic rhinitis–OR = 1.7, atopic dermatitis–OR = 2.3 and allergic sensitization–OR = 1.5.

Calculations were performed with *Statistica* package.

## Results

### The FLG R501X variant is rare in a Polish population

While analyzing the 3802 subjects from ECAP cohort we identified 3629 wild type, 140 heterozygous and three homozygous 2282del4 genotypes (carriage rate: 3.76%, CI: 3.20–4.41) as well as 30 heterozygous R501X genotypes (carriage rate: 0.8%, CI: 0.55–1.12). There were no compound heterozygotes. In order to verify relatively low frequency of R501X vs. 2282del4 variant in a Polish population we also tested 510 samples randomly selected from an anonymous bank containing DNA isolated for the purpose of paternity tests [16]. We found 19 samples positive for 2282del4 (carriage rate: 3.79%, CI: 2.44–8.85) and five positive for R501X (carriage rate: 1.0%, CI: 0.43–2.31). The distribution of *FLG* variants did not deviate from Hardy-Weinberg equilibrium in either cohort (P = 0.5 in both cases).

### Associations between FLG variants and studied phenotypes

When analyzing distribution of combined *FLG* variants we found an association with AD (OR = 2.01, P = 0.007), asthma (OR = 1.70, P = 0.024) and AR (OR = 1.43, P = 0.046, Table 1). Analysis of individual variants showed statistically significant associations between 2282del4 and AD (OR = 1.92, P = 0.022), asthma (OR = 1.97, P = 0.005), and AR (OR = 1.47, P = 0.047, Table 1).

**Table 1:** Prevalence of *FLG* variants according to clinical diagnosis.

		2282del4			R501X			2282del4 or R501X		
Diagnosis	n	n (%)	OR (95%CI)	P	n (%)	OR (95%CI)	P	n (%)	OR (95%CI)	P
**Atopic Dermatitis**	271	16 (5.9)	1.92 (1.09–3.39)	0.022	4 (1.5)	2.31 (0.74–7.22)	0.14	20 (7.4)	2.01(1.20–3.36)	0.007
**Asthma**										
All	414	25 (6.0)	1.97 (1.22–3.18)	0.005	1 (0.2)	0.37 (0.05–2.89)	0.3	26 (6.3)	1.70 (1.07–2.69)	0.024
Atopic	186	14 (7.5)	2.49 (1.36–4.56)	0.002	1 (0.5)	0.84 (0.11–6.46)	0.9	15 (8.1)	2.22 (1.24–3.96)	0.006
Non-atopic	228	11 (4.8)	1.55 (0.8–3.0)	0.19	0	NA	0.2	11 (4.8)	1.28 (0.67–2.46)	0.5
**Allergic Rhinitis**										
All^1^	1114	51 (4.6)	1.47 (1.01–2.15)	0.047	8 (0.7)	1.22 (0.49–3.04)	0.7	59 (5.3)	1.43 (1.01–2.04)	0.046
persistent	591	33 (5.6)	1.81 (1.17–2.8)	0.007	4 (0.7)	1.05 (0.34–3.28)	0.9	37 (6.3)	1.69 (1.12–2.54)	0.011
intermittent	497	17 (3.4)	1.08 (0.63–1.88)	0.7	4 (0.8)	1.37 (0.43–4.31)	0.59	21 (4.2)	1.31 (0.69–1.86)	0.63
Healthy controls	1865	59 (3.2)			12 (0.6)			71 (3.8)		

1 In 24 subjects allergic rhinitis could not be classified as intermittent or persistent; NA not applicable; All comparisons vs. healthy controls.

Further analysis indicated that the statistically significant associations between 2282del4 or combined genotype and asthma as well as AR were limited, respectively, to atopic asthma (AA) and persistent AR (pAR, Table 1).

We noted that when subjects were stratified according to the answer to the question: ‘Have you ever had asthma’ the frequency of *FLG* variants was statistically significantly increased only among those who were diagnosed with asthma by a physician during the present study but who were not aware of having the disease as judged by questionnaire data (OR = 1.81, P =  0.03 and OR =  3.53, P = 0.00005, for the comparison of combined genotype frequency vs. healthy controls, for all asthma and AA, respectively, Table 2). Had the study been based solely on questionnaire data, only a trend for association between combined *FLG* variants and asthma would have been found (OR = 1.15, P = 0.83, Table 2).

**Table 2:** Prevalence of the *FLG* variants vs. concordance between diagnosis of asthma (all kinds or atopic asthma) by a physician (i.e. diagnosed during the present study) and individual awareness of having asthma (all kinds) according to questionnaire data.

Diagnosis:			2282del4	R501X	2282del4 or R501X	OR (CI) P *
Physician	Questionnaire	N	n (%)	n (%)	n (%)	
*All asthma*						
+	+	127	7 (5.5)	0	7 (5.5)	1.47 (0.68–3.22) 0.34
+	-	269	17 (6.3)	1 (0.4)	18 (6.7)	**1.81 (1.07–3.07) 0.03**
*Atopic asthma*						
+	+	61	2 (3.3)	0	2 (3.3)	0.86 (0.23–3.25) 0.9
+	-	116	12 (10.3)	1 (0.9)	13 (11.2)	**3.53 (1.86–6.72) 0.00005**
*Asthma (all) by questionnaire irrespective of physician's diagnosis*		206	9 (4.4)	0	9 (4.4)	1.15 (0.58–2.32) 0.83
Healthy controls		1865	59 (3.2)	12 (0.6)	71 (3.8)	

* Calculated for the comparison of the frequency of the combined genotype (2282del4 or R501X) vs. healthy controls. Cells with P values <0.05 are **boldfaced**; Questionnaire data were not available in 18 subjects with asthma including 9 with AA.

In ECAP cohort there was no association between *FLG* variants and allergic sensitizations or total IgE concentration. The prevalence of the combined genotype was 4.7% (78/1676) vs. 4.5% (95/2126) among those with a positive skin-prick test to at least one allergen and the remaining group, respectively (OR = 1.04, CI:0.77–1.42, P = 0.8). Mean concentration of total IgE was 9.08 (CI: 7.38–11.17) vs. 9.62 (CI: 9.20–10.06) for those with and without *FLG* defects, respectively (P = 0.6, analysis adjusted for test-method, sex and age category).

### The association between FLG null variants and atopic asthma (AA) is also found among those without atopic dermatitis (AD) or history thereof

Although the OR for AA conferred by 2282del4 or the combined genotype was higher among those with than those without AD (OR = 4.37 vs. OR = 2.23, and OR = 3.61 vs. OR = 2.02, respectively) the associations were statistically significant in both subgroups (P<0.03, Table 3). Analysis of pAR showed similar trends although the statistical significance of these associations among those without AD was borderline (P = 0.049 and P = 0.053, for 2282del4 and combined *FLG* variants, respectively (Table 4).

**Table 3:** Distribution of the *FLG* variants in subjects with atopic asthma (AA) stratified by diagnosis of atopic dermatitis.

		2282del4			R501X			Combined genotype		
Atopic dermatitis	n	n (%)	OR (CI)	P	n (%)	OR (CI)	P	N (%)	OR (CI)	P
-	162	11 (6.8)	2.23 (1.15–4.33)	0.015	1 (0.6)	0.96 (0.12–7.42)	0.97	12 (7.4)	2.02 (1.07–3.81)	0.027
+	24	3 (12.5)	4.37 (1.27–15.07)	0.011	0	NA	0.69	3 (12.5)	3.61 (1.05–12.38)	0.029

All comparisons vs. healthy controls (Table 1), NA: not available.

**Table 4:** Distribution of the *FLG* variants in subjects with persistent allergic rhinitis (pAR) stratified by diagnosis of atopic dermatitis.

		2282del4			R501X			Combined genotype		
Atopic dermatitis	n	n (%)	OR (CI)	P	n (%)	OR (CI)	P	n (%)	OR (CI)	P
-	523	26 (5.0)	1.6 (1.0–2.57)	0.049	4 (0.8)	1.19 (0.38–3.71)	0.76	30 (5.7)	1.54 (0.99–2.38)	0.053
+	68	7 (10.3)	3.51 (1.54–8.01)	0.001	0	NA	0.51	7 (10.3)	2.9 (1.28–6.57)	0.008

All comparisons vs. healthy controls (Table 1), NA: not available.

The frequency of the combined genotype showed a trend for increase among subjects who were not diagnosed with AD but who reported history of an inflammatory skin condition in the questionnaire (OR = 1.38, CI: 0.96–2.0, P = 0.08, comparison vs. healthy controls). Thus, we were interested whether the observed associations between *FLG* variants and AA among those without AD could be caused by an association among those with a history of AD or other inflammatory skin disease. However, this was not apparent since there was an association between 2282del4 or combined genotype and AA also among subjects without AD according to both clinical diagnosis and self reported history of an inflammatory skin condition: 7.1% (7/99) vs. 3.1% (55/1790), OR = 2.40 (CI: 1.06–5.42), P = 0.03 and 8.1% (8/99) vs. 3.7% (66/1790), OR = 2.30 (CI: 1.07–4.93), P = 0.03, for 2282del4 and combined genotype, respectively.

Since the chances of AD resolution increase with age we also analyzed the association between AA and *FLG* variants among those without AD in the youngest age group (i.e. children 6–7 y.o.). Among those with AA the prevalence of 2282del4 and the combined genotype was 12.9% (4/31) which was higher than among controls (OR =  4.53, CI: 1.54–13.38, P = 0.018 and OR = 3.74, CI: 1.28–10.98, P = 0.032, for 2282del4 and the combined genotype, respectively).

Conversely, analysis of pAR did not show associations with *FLG* variants among those without AD according to both the questionnaire and clinical diagnosis: 3.8% (13/340) vs. 3.1% (55/1790), OR = 1.25 (CI: 0.68–2.32, P = 0.5), and 4.7% (16/340) vs. 3.7% (66/1790), OR = 1.29 (CI: 0.74–2.26, P = 0.4) for prevalence of 2282del4 and the combined genotype among pAR and healthy controls, respectively.

## Discussion

While studying a population based cohort of subjects we observed that the *FLG* defects conferred an increased risk for development of AD, AR (in particular pAR) and asthma (in particular AA). Whereas both associations were particularly strong among subjects with AD, the association with AA remained after exclusion of subjects with current AD even when analysis was limited to the youngest age group, i.e. a group with the lowest chance of complete resolution of skin disease. Association between AA and *FLG* variants was also present among those without current AD or history of AD or other inflammatory skin disease.

The association between *FLG* defects and AA in the absence of AD contrasts with conclusions of two recent meta-analyses although it should be noted that both these studies reported trends in the direction of association (OR = 1.11 and OR = 1.30) [5], [6].

On one hand, some cases of resolved AD might have been missed in our study due to lack of patients'/parents' recall. Recall errors regarding history of allergic diseases have been demonstrated [17] and are likely to exist also in our cohort. On the other hand, the discrepancy with pervious studies [5], [6] might also be caused by population specific genetic, environmental and/or life style factors as well as methodological issues. In our cohort the association between *FLG* null variants and asthma (in particular AA) was found preferentially (exclusively?) among those who were not aware of having the disease. This suggests that AA associated with *FLG* defects in the absence of AD may have a subtle phenotype being particularly difficult to diagnose by family practitioners. Notwithstanding the precise reasons for the discussed discrepancies, our results indicate that in a Polish population *FLG* defects represent a risk factor for asthma, irrespective of apparent skin disease or history thereof which it is possible to elicit in a clinical setting.

Interestingly, association between *FLG* defects and asthma without eczema has also been found in a cohort of Danish children prospectively followed from birth [18]. Furthermore, similar longitudinal follow-up methodology which should maximize the diagnosis rate was also employed in a study of German cohort where a relatively distinct trend towards an association was found (OR = 2.47, P = 0.11) [8]. Further evidence implicating epidermal barrier function in asthma pathogenesis came recently from a large genome-wide study showing that a locus with a likely function in keratinocytes (*RORA*) was among ten loci most strongly associated with this disease [19]. These findings suggest that at least in some cohorts epidermal barrier defects may play a role in asthma pathogenesis among those without AD.

In contrast to studies in other populations [5], [6] we did not observe an association between *FLG* null variants and allergic sensitization(s) as judged by analysis of skin prick test results or concentration of total IgE. This result is consistent with recent observations in a Danish cohort where the risk of sensitization among *FLG* defect carriers increased only after onset of asthma and/or eczema [18] and suggests that the effect of *FLG* null variants on AD or AA development is not likely to be primarily mediated through allergic sensitization.

The association between *FLG* variants and AD confirms the findings in other populations [3], [6]. However, the association found in our study had only moderate statistical significance and effect size. This is consistent with suggestions that *FLG* variants are associated with severe forms of AD which are more readily ascertained in hospital based studies [20]–[23].

Our results also add to data on differences in population specific prevalence of *FLG* variants. We showed that in a Polish population the prevalence of the R501X variant (∼1%) was distinctly lower than the prevalence of ∼6% reported for Irish and Scottish populations [2]. An intermediate R501X frequency in German population (∼2.5% as estimated from pooled data of Stemmler et al. [24] and Weidinger et al. [20]) suggests a clinal variation in prevalence of this variant in Europe.

In conclusion, we show that in a Polish population *FLG* null variants 2282del4 and R501X are risk factors for AD, and independently from it, for AA. A methodological observation is that in a Polish population AA associated with *FLG* defects may have a subtle phenotype being difficult to diagnose by a questionnaire.
